# *Morganella morganii*: An unusual analysis of 11 cases of pediatric urinary tract infections

**DOI:** 10.1002/jcla.24399

**Published:** 2022-03-29

**Authors:** Huixuan Shi, Xianrui Chen, Yonghua Yao, Jinping Xu

**Affiliations:** ^1^ 117892 Department of Pediatrics The First Affiliated Hospital of Xiamen University Xiamen China; ^2^ Pediatric Key Laboratory of Xiamen Xiamen China; ^3^ Institute of Pediatrics School of Medicine Xiamen University Xiamen China

**Keywords:** bacterial infections, children, community‐acquired, *Morganella morganii*, urinary tract infection

## Abstract

**Background:**

The increase in rare opportunistic microbial infections caused by *Morganella morganii* is alarming across the globe. It has been reported that in cases of urinary tract infections (UTIs) caused by *M. morganii*, however, few studies investigated children. Our study aimed to analyze the risk factors, antimicrobial susceptibility, and clinical characteristics, so as to improve the clinical diagnosis and therapy of *M. morganii* infection.

**Methods:**

Between April 1, 2017 and April 1, 2021, 11 cases of pediatric UTIs caused by *M. morganii* were included in this retrospective study. Medical records were reviewed and analyzed.

**Results:**

The study population included 10 males and one female between 11 months and 13 years old (mean age: 4 years 9 months). The most common comorbidity was nephrotic syndrome (72.7%, 8/11). Six patients (54.5%) were in the immunosuppressed state due to chemotherapy or immunosuppressant therapy. Ten cases defined as lower UTIs with no specific clinical manifestations had normal or slightly elevated leukocyte counts and procalcitonin (PCT) levels, and normal C‐reactive protein (CRP) levels. One child diagnosed upper UTIs accompanied with fever, high level of leukocyte counts, CRP, and PCT. The *M. morganii* presented 100% susceptibility to aztreonam, ertapenem, meropenem, piperacillin/tazobactam, cefepime, ceftazidime, cefotetan, ticarcillin/clavulanic acid, and cefoperazone/sulbactam. Almost all patients had good responses to third‐generation cephalosporins antibiotic therapy.

**Conclusion:**

Clinical vigilance for the possibility of *M. morganii* in pediatric UTIs in combination with underlying disease or immunosuppression is warranted. Treatment strategies should be proposed according to the clinical condition and the antibiotic susceptibility results.

## Graphical abstract

In this prospective study, urinary tract infections (UTIs) caused by *M. morganii* among children had good responses to third generation cephalosporins antibiotic therapy. Continuous monitoring of antimicrobial resistance trends of *M. morganii* are necessary to inform paediatricians aiming at reducing the associated morbidities and mortalities.

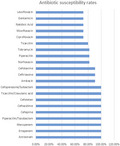

## 1 INTRODUCTION

*Morganella morganii* is a gram‐negative, rod‐shaped, and facultative anaerobic bacillus, which belongs to human gut commensal microbiota.^1^ It is considered as a non‐negligent opportunistic pathogen that mainly causes various infections, such as sepsis, abscess, urinary tract infections (UTIs), chorioamnionitis, and cellulitis.^2^ Furthermore, on rare occasions, it may cause potentially fatal systemic infection, especially in postoperative environment and nosocomial as well as in young children and patients with impaired immune system.^1^ UTIs are among the most common bacterial infections in children and are associated with significant short‐ and long‐term morbidity.^3^, ^4^ Precise treatment decisions help to prevent chronification of UTIs in children, related the development of antibiotic resistance, voiding dysfunctions, renal scarring, and systemic abiosis. Uropathogens involved in UTIs should be identified with precision to allow targeted therapeutic decisions.^5^ In recent years, more and more published clinical case reports have attempted to clarify the clinical manifestation and management strategies of UTIs caused by *M. morganii*.^6^, ^7^ *M. morganii* is recognized as a new clinical treatment challenge because of ongoing acquisition of antimicrobial resistance genes, which may bring more extensive and challenging multidrug resistance issue.^8^ The first study investigating community‐acquired UTIs caused by *M. morganii* in children has been reported in Turkey.^9^ Our study aims to explore the risk, antimicrobial susceptibility, and clinical characteristics, so as to improve the treatment and prognosis of children with UTI caused by *M. morganii*.

## 2 MATERIALS AND METHODS

### 2.1 Samples

From April 1, 2017 to April 1, 2021, a total of 31,705 children were admitted and treated in the pediatric department of the First Affiliated Hospital of Xiamen University. Among the 625 patients proven UTIs, 11 cases with *M. morganii* UTIs were retrospectively evaluated. The medical records of children diagnosed with UTIs caused by *M. morganii* were reviewed. Patient information, including demographics, medical history, laboratory findings, treatments, and outcomes were summarized and analyzed.

### 2.2 Instruments and reagents

Urine specimens may have originated from clean‐catch midstream urine or aseptic bladder catheterizations. Urine samples were inoculated onto eosin methylene blue agar (Autobio Diagnostics Co Ltd.) and Colombian blood agar medium (Autobio Diagnostics Co Ltd.). All inoculated plates were incubated in Thermo M3111 incubator (Thermo Inc.). Pathogen identifications were performed using the Vitek MS‐CHCA (BioMérieux Inc.) and Vitek‐MS automated microbial identification system (BioMérieux Inc.). The cut‐off used for significant bacterial presence was ≥1000–50,000 colony‐forming units (CFU)/ml for urine specimen from bladder catheterization (≥10^4^ CFU/ml with symptoms or ≥10^5^ CFU/ml without symptoms for urine specimen from midstream void).^10^

BioMerieux mini Vidas automated immunoassay analyzer (BioMérieux Inc.) and procalcitonin (PCT) kit were used to detect serum PCT. C‐reactive protein (CRP) was detected by VITROS 5,1 FS analyzer (Ortho‐Clinical Diagnostics) using the manufacturer's reagents. The new UF‐1000i analyzer (Sysmex) was used for urinalysis. The urinalysis with positive leucocyte esterase and/or nitrite, bacteriuria, or pyuria is highly sensitive for UTI.

### 2.3 Antimicrobial susceptibility testing

Antimicrobial susceptibility testing of *M. morganii* isolates was determined by the automated VITEK 2 compact microbiology analyzer (BioMérieux Inc.) using antimicrobial susceptibility testing (AST)‐GN67 and XN04 cards (BioMérieux Inc.). Results were interpreted as sensitive, intermediate, and resistance based on the Clinical and Laboratory Standards Institute's (CLSI) criterias.^11^, ^12^, ^13^, ^14^, ^15^ The following antimicrobial agents were used at the concentrations shown: amikacin (30 µg), ampicillin/sulbactam (10/10 µg), amtronam (30 µg), ertapenem (10 µg), trimethoprime/sulfamethoxazole (1.25/23.75 µg), ciprofloxacin (5 µg), fosfomycin (200 µg), chloramphenicol (30 µg), meropenem (10 µg), minocycline (30 µg), moxifloxacin (5 µg), naproxic acid (30 µg), norfloxacin (10 µg), piperacillin (100 µg), piperacillin/tazobactan (100/10 µg), gentamicin (10 µg), ticacillin (75 µg), ticacillin/clavulanic acid (75/10 µg), cefepime (30 µg), cefuroxime (30 µg), cefoperazone/sulbactam (75/30 µg), ceftriaxone (30 µg), cephalothifen (30 µg), cefotaxime (30 µg), ceftazidime (30 µg), cefotetan (30 µg), cefazolin (30 µg), cefazoxime (30 µg), tobramycin (10 µg), imipenem (10 µg), levofloxacin (5 µg), amoxicillin/potassium clavulanate (20/10 µg), ampicillin (10 µg), furantoin (300 µg), tetracycline (30 µg), and tigecycline (15 µg). For statistical analysis purpose, “intermediate” sensitivity results of bacterial isolates were grouped to “resistant” sensitivity results. Multi‐drug resistance was defined as resistance of an isolate to three or more antimicrobial classes tested.^16^

### 2.4 Quality control

The quality control strains of pathogen identification were *E. coli ATCC 8739* strains (Biomerieux Inc.). For quality control of susceptibility tests, *E. coli ATCC25922* strains (National Center for Clinical Laboratories) were used.

### 2.5 Ethical clearance

The study was approved by Ethical Review Committee of the First Affiliated Hospital of Xiamen University with number [2022] ERSR‐012.

## 3 RESULTS

In the 625 patients proven UTI, only 11 cases were identified *M. morganii* positive in clean‐catch midstream urine culture. During the same period, *M. morganii* was not detected in the cultures of other body fluids (sputum, cerebrospinal fluid, broncho‐alveolar lavage fluid, pleural effusion, peritoneal effusion, secretion, or exudate, etc.) and catheter culture (central venous catheter, tracheal catheter, thoracic drainage tube, abdominal drainage tube, etc.). The study population included 10 males and one female between 11 months and 13 years old (mean age: 4 years 9 month). The most common comorbidity was nephrotic syndrome (72.7%, 8/11), though systemic lupus erythematosus (SLE) (1/11), acute lymphocytic leukemia (ALL) (1/11), and left ureteropelvic junction obstruction (1/11) also occurred. Six patients (54.5%) were in the immunosuppressed state due to chemotherapy or immunosuppressant therapy. Only one patient (case 6) had fever and none of the patients had urinary tract irritation symptoms. The *M. morganii* strains were isolated from the samples collected for the culture and urinalysis at the first day of hospitalization. At the time of presentation, all patients had no central venous catheter or urinary catheter. (The characteristics and the clinical manifestation of patients are shown in Table 1). Ten cases defined as lower UTIs with no specific clinical manifestations observed had normal or slightly elevated leukocyte counts and PCT levels, and normal CRP levels. No other bacterial coinfection was isolated from all the urine and blood cultures. Only one child (case 6) diagnosed with upper UTIs accompanied high level of white blood cell (WBC) count, CRP, and PCT. Urinalyses were positive for leukocytes in 11 (100%), hematuria in 3 (36.4%), nitrite in 1 (9.1%), and bacteriuria in 7 (63.6%) children. The laboratory findings were presented in Table 2.

**TABLE 1:** Characteristics and the clinical manifestation of patients complicated with *Morganella morganii*

Case	Gender	Age	Comorbidity	Use immunosuppressant/chemotherapy drugs	Medical history and cause of admission
1	Male	7 years	Nephrotic syndrome	None	Edema for 10 days
2	Male	1 year and 8 months	Nephrotic syndrome	None	Eyelid edema for 12 days and abnormal urine test for 7 days
3	Male	5 years and 10 months	Nephrotic syndrome	Prednison	Nephrotic syndrome for 6 months, urine protein positive accompanied by cough for 10 days
4	Male	2 years and 4 months	Nephrotic syndrome	None	Edema for 18 days and cough for 3 days
5	Male	13 years	SLE and lupus nephritis	Prednisolone, Mycophenolate Mofetil	SLE for 1 year, with tawny urine for 1 month and abdominal pain for 3 days
6	Male	11 months	Left ureteropelvic junction obstruction	None	Fever for 1 day with abnormal urine test
7	Male	2 years and 8 months	Nephrotic syndrome	Prednisone	Systemic edema for 8 days and abnormal urine test for 5 days
8	Male	2 years and 6 months	Nephrotic syndrome	Prednisone	Nephrotic syndrome for 1 months, urine protein positive for 1 day
9	Male	3 years and 9 months	ALL	Chemotherapy	ALL for 9 months and hospitalized for chemotherapy
10	Female	9 years	Nephrotic syndrome	Prednison	Nephrotic syndrome for 2 years, urine protein positive for 2 days
11	Male	3 years and 7 months	Nephrotic syndrome	None	Cough for half a month, eyelid edema for 10 days

Abbreviations: ALL, acute lymphocytic leukemia; SLE, systemic lupus erythematosus.

**TABLE 2:** Laboratory findings of patients

Case	WBC count (10^9^/L)	Neutrophil (%)	CRP (mg/L)	PCT (ng/ml)	Blood culture and other bacterial infections	WBC count of urinalysis (/µl)	Bacteriua of urinalysis (/µl)	RBC count of urinalysis (/µl)	Nitrite of urinalysis	B ultrasonography of urinary system	Infection time
1	10.93	59.2	7	0.464	Negative	38	2.8	5	Negative	Bilateral renal enlargement	2017/04/08
2	6.56	17.3	<0.499	0.107	Negative	54.1	46.9	5.8	Negative	Normal	2018/01/04
3	11.6	80.2	0.89	0.049	Negative	77	483	1.5	Negative	Normal	2018/09/04
4	14.16	59.5	<0.499	0.112	Negative	59.8	38.7	2.9	Negative	Normal	2019/10/23
5	7.5	61.27	4.6	0.074	Negative	64.5	34.1	31.9	Negative	Normal	2019/11/17
6	13.73	43.5	156.64	1.59	Negative	6454.7	45.5	14.4	Negative	Left kidney hydronephrosis	2019/02/08
7	11.03	25.4	<0.499	0.266	Negative	70.4	11.3	0.7	Negative	Normal	2019/04/03
8	8.68	48.6	<0.499	0.042	Negative	66.3	11.3	1.1	Negative	Normal	2020/01/14
9	3.18	37.5	<0.499	/	Negative	242	>99,999	2.9	Positive	Normal	2020/05/25
10	7.5	52.3	2	0.211	Negative	56	240	29.4	Negative	Normal	2020/09/03
11	6.34	56.9	<0.499	0.12	Negative	43.7	4.6	29.6	Negative	Normal	2021/03/12

Note

Reference intervals were the following: CRP (0–10 mg/L), PCT (<0.046 ng/ml, WBC count of urinalysis [0–18/µl]), RBC count of urinalysis (0–15/µl), bacteriua of urinalysis (0–11.4/µl).

Abbreviations: CRP, C‐reactive protein; PCT, procalcitonin.

Susceptibility test results of *M. morganii* isolates against various antibiotics were shown in Table S1. The *M. morganii* presented 100% susceptibility to aztreonam (11/11), ertapenem (11/11), meropenem (11/11), piperacillin/tazobactam (11/11), cefepime (11/11), ceftazidime (11/11), cefotetan (10/10), ticarcillin/clavulanic acid (7/7), and cefoperazone/sulbactam (6/6). The *M. morganii* was almost sensitive to amikacin (90.9%, 10/11), ceftriaxone (10/11) and cefotaxime (10/11), and norfloxacin (81.8%, 9/11). The susceptibility testing demonstrated 72.7% (8/11) susceptibility to ciprofloxacin, moxifloxacin, nalidixic acid, gentamicin, ticarcillin, levofloxacin, but 100% resistance to cefuroxime axetil (11/11), cefalotin (11/11), cefazolin (3/3) and 90.9% to ampicillin/sulbactam (10/11).

Almost all patients (10/11) had good responses to third‐generation cephalosporins antibiotic therapy according to guideline.^4^ Furthermore, one child (case 5) withdrew treatment because of economic problem. None of the children developed any complications including sepsis, shock, meningitis, etc. The clinical information of 11 patients with *M. morganii* infection was presented in Table 3.

**TABLE 3:** Clinical information of patients

Case	Antibiotics before urine culture results	Antibiotics after urine culture results	Duration of effective antibiotics therapy	Length of stay	Outcome of infection
1	Flucloxacillin (3 days)	Cefotaxime (3 days)	3 days	11 days	Cure
2	None	Cefixime (3 days)	3 days	12 days	Cure
3	Cefotaxime (5 days)	Ceftazidime (3 days)	3 days	11 days	Cure
4	Cefaclor (3 days)	Ceftriaxone (3 days)	3 days	27 days	Cure
5	None	Withdrawing treatment	Loss to follow‐up	2 days	Loss to follow‐up
6	Cefixime (1 day)	Cefotaxime (10 days)and cefixime (3 days)	14 days	13 days	Cure
7	Amoxicillin/clavulanate potassium (5 days)	Ceftriaxone (7 days)	7 days	13 days	Cure
8	None	Cefixime (5 days)	5 days	9 days	Cure
9	Compound sulfamethoxazole (8 months)	Cefixime (3 days)	3 days	2 days	Cure
10	Metronidazole and amoxicillin/potassium clavulanate (2 days)	Cefixime (3 days)	3 days	13 days	Cure
11	Amoxicillin/clavulanate potassium (3 days)	Cefixime (3 days)	3 days	8 days	Cure

## 4 DISCUSSION

*Morganella morganii* has been considered a clinical and *community*‐acquired pathogen with opportunistic infections detected in diverse organ infections across all age groups globally.^17^ *M. morganii* has been reported causing various infections such as bacteremia, osteomyelitis, and UTIs. Claire et al. reported a 3‐year‐old girl with hematologic malignancy suffering from *M. morganii* bacteremia was successfully treated with ceftazidime‐avibactam and aztreonam combination.^18^ *M. morganii* was cultured positive in blood sample and cephalohematoma aspirated from a neonate developing an infected right parietal cephalohematoma and underlying osteomyelitis.^6^ The previous studies of UTIs caused by *M. morganii* were almost reported in adult. In Jan Hrbacek et al.’s^19^ survey from Central European Urology Department between 2011–2019, *M. morganii* (n = 194, 26.2%) was positive in the pathogens surveyed in urine cultures. Leyla et al. (2019)^20^ reported three *M. morganii* strains were isolated from the urine samples of patients with community‐acquired UTIs. In 2020, a study from Turkey presented 11cases of community‐acquired UTIs caused by *M. morganii* in children.^9^ Similar studies have not been conducted in China.

Compared with the only other pediatric study in Turkish population,^9^ we had observed some differences and similar results in these two samples. The clinical manifestations of UTIs in children are nonspecific and largely depend on the age. UTIs in newborn and young infants always manifest as diverse and non‐specific symptoms including temperature instability, hyperexcitability, jaundice, lethargy, abdominal pain, irritability, etc.^10^ In older children, the manifestations are more specific including irritative voiding symptoms, fever, chillness, vomiting, abdominal pain, and costovertebral knocking pain, etc.^21^ In the Turkish population,^9^ irritability (n = 5, 45.5%) and dysuria (n = 5, 45.5%) were the most frequent symptoms of infants with UTIs. Nevertheless, almost all the children had no specific clinical symptoms except for one child with fever in our research. In both samples, none of the patients developed renal failure or electrolyte abnormalities and had clinical signs of bacteremia/sepsis. Of the 11 Turkish patients with urine studies, 81.8% had leukocyturia and 45.5% had hematuria. Similarly, the urinalysis which was positive for leukocytes and hematuria in our sample was 100% and 36.4%, respectively. Nitrite test has a low sensitivity (about 50%) but high specificity (98%) for pediatric UTIs.^21^ However, children with positive nitrite was 9.1% (1/11) in our study compared to 54.5% (6/11) in the Turkish study. Atm B et al.^9^ showed that 45.5% of children had normal CRP and leukocyte levels in UTI caused by *M. morganii*. In our study, 10 cases with lower UTIs had normal or slightly elevated leukocyte counts and PCT levels, and normal CRP levels. Only one child (case 6) diagnosed upper UTIs accompanying high level of WBC, CRP, and PCT had left kidney hydronephrosis. Our results were in accord with past reports indicating that CRP had a lower specificity for identifying children with renal parenchymal involvement, whereas a cut‐off value of 1.0 ng/ml of PCT had been shown to be predictive of acute pyelonephritis in young children.^22^

The previous investigations had revealed risk factors for *M. morganii* infections including old age, repeat hospitalizations, nursing home residence, urethral catheter, and prematurity, etc.^23^, ^24^ None of the children enrolled in our study underwent invasive procedure including peripheral central vein catheterization, urethral catheterization or other operation, which was in accord with the study of Turkish population.^9^ *M. morganii* infections were frequently associated with a history of immunocompromised condition or immunodeficiency diseases such as acquired immune deficiency syndrome, and X‐linked agammaglobulinemia.^17^, ^25^ In accordance with previous research features, six patients (54.5%) in our study were in the immunosuppressed state due to chemotherapy or immunosuppressant therapy. Unlike the Turkish research,^9^ all of our patients have comorbidities, the most common of which is nephrotic syndrome (72.7%, 8/11). The causes of UTIs in nephrotic children include a low level of serum IgG, hypoproteinemia, decreased bactericidal activity of the leukocytes, declined perfusion of the spleen, corticosteroids treatment, and loss of properdin in the urine.^26^ *M. morganii* should be placed as a clinically significant pathogen in the causative possibilities of UTIs in nephrotic children. One patient was detected left ureteropelvic junction obstruction which is an established risk factor for recurrent UTIs. However, there is no convincing evidence that malformation in the urinary system maybe a risk factor for *M. morganii* infection.

Various mechanisms including intrinsic, acquired, and adaptive resistances can lead to antibiotic resistance. *M. morganii* has intrinsic resistance to amoxicillin, oxacillin, most of the first‐ and second‐generation cephalosporins, ampicillin, macrolides, lincosamides, glycopeptides, fosfomycin, fusidic acid, and colistin.^2^ Jan Hrbacek et al.^19^ detected antibiotic susceptibility of 194 *M. morganii* strains from urine cultures and revealed the cumulative resistance rates of ampicillin (95.7%), amoxiillin/clavulanic acid (92.9%), cefuroxime (89.3%), and nitrofurantoin (95.9%). High resistance to cefuroxime axetil (100%), cefalotin (100%), cefazolin (100%), amoxicillin/clavulanate potassium (100%), nitrofurantoin (100%) and ampicillin/sulbactam (90.9%) was observed in our study. Multidrug‐resistant *M. morganii* strains carrying mobile genetic elements including plasmids integrons have been presented in the literatures. Fatimah et al.^27^ identified that *M. morganii* (n = 13) counted for 3.7% of extended‐spectrum beta‐lactamase (ESBL) infections and modest‐to‐high resistant to tigecycline (6/13). In the study of N. Mbelle et al.,^28^ they detected that two multidrug‐resistant *M. morganii* strains from UTIs were only susceptible to carbapenems, amikacin, and tigecycline. Xiaobing Guo et al.^29^ also confirmed that *M. morganii L241* exhibited resistance to almost all of the β‐lactam antibiotics including piperacillin/tazobactam, cefotaxime, ceftazidime, cefepime, cefpirome, ertapenem, imipenem, and meropenem with the exception of aztreonam. Nevertheless, there is no multidrug‐resistant *M. morganii* strain observed in our research. More attention should be paid to the multi‐drug resistance and extensively drug‐resistant *M. morganii* strains which are increasing globally resulting in treatment failure.^17^

*M. morganii* is normally susceptible to aztreonam, aminoglycosides, antipseudomonal penicillins, third‐ and fourth‐generation cephalosporins, carbapenems, quinolones, trimethoprim/sulfamethoxazole and chloramphenicol.^2^ Atm et al.,^9^ who described antibiotic resistance of *M. morganii* in pediatric UTIs, reported 100% susceptibility to imipenem, meropenem, piperacillin‐tazobactam and 90.9% to amikacin. Compared to the data of Turkish study,^9^ our study proved the same susceptibility rate of meropenem, piperacillin‐tazobactam and amikacin, but susceptibility rate of imipenem (44.4%, 4/9) was lower in our survey. Although the exact reasons for conflicting results of imipenem susceptibility are unclear, differences in age, race, geographic area, and methodology may explain the discrepancies. The antibiotic susceptibility rates of an adults’ UTIs investigation enrolling 194 strains of *M. morganii* were compared to our results including meropenem (98.9% vs. 100%), amikacin (95.7% vs. 90.9%), ertapenem (86.8% vs. 100%), piperacillin/tazobactam (86.4% vs. 100%), ceftazidime (86.3% vs. 100%), cefepime (85.7% vs. 100%), cefotaxime (83.7% vs. 90.9%), ofloxacin (73.9% vs. 72.7%), cotrimoxazole (72.5% vs. 63.6%), and ciprofloxacin (65.6% vs. 72.7%).^19^ The differences may be due to the small sample size of our study group and different clinical features between children and adults. For the treatment of *M. morganii* infections, gentamicin was the most frequently used antibiotic, and ciprofloxacin, amikacin, and ceftriaxone were also used frequently in adult patients, but the use of the third‐generation cephalosporins is still recommended in the children in the consideration of the aminoglycoside‐induced nephrotoxicity or cochleotoxicity.^30^ In Turkish study, hospitalized children received piperacillin‐tazobactam, whereas outpatients were given amikacin, with good clinical response.^9^ In our study, almost all patients (10/11) had good responses to third‐generation cephalosporins antibiotic treatment. None of the children developed any complications including sepsis, shock, meningitis, etc.

To our knowledge, this study firstly described the clinical manifestations and resistance patterns of *M. morganii* in pediatric UTIs in China. However, our research has certain limitations. Firstly, this was a single‐center study with the limitation of sample size. Additionally, the manifestations related information was limited in our retrospective study.

In summary, our study described that lower UTIs caused by *M. morganii* with no specific clinical manifestations had normal or slightly elevated leukocyte counts and PCT levels, and normal CRP levels, but pyelonephriti may accompany with fever symptom, high level of WBC, CRP, and PCT. Immunocompromised condition is one of *M. morganii* infection risk factors. The *M. morganii* presented high susceptibility to aztreonam, ertapenem, meropenem, piperacillin/tazobactam, cefepime, ceftazidime, cefotetan, ticarcillin/clavulanic acid, and cefoperazone/sulbactam. Almost all patients had good responses to third‐generation cephalosporins antibiotic treatment. Most UTIs caused by *M. morganii* patients have a good prognosis with no further complications.

*Morganella morganii* is a conditionally pathogenic microorganism with relatively few cases of clinical infection among children. Clinical vigilance for the possibility of *M. morganii* in pediatric UTIs in combination with underlying disease or immunosuppression is warranted. Continuous monitoring of antimicrobial resistance trends of *M. morganii* is necessary to inform paediatricians aiming at reducing the associated morbidities and mortalities. Treatment strategies should be proposed according to clinical condition and the antibiotic susceptibility results.

## CONFLICT OF INTEREST

The authors declare no conflict of interest.

## AUTHOR CONTRIBUTIONS

Chen Xianrui and Shi Huixuan designed the study. Shi Huixuan analyzed and interpreted the data, and drafted the article. Yao Yonghua and Xu Jinping acquired the data. Chen Xianrui is the corresponding author who contributed to conception and design, critical revision of the article for important intellectual content. All authors read and approved the final manuscript.

## Supporting information

Table S1

## Data Availability

The data that support the findings of the current study are available from the corresponding author upon reasonable request.
