# Supplementary material for: Morganella morganii: An unusual analysis of 11 cases of pediatric urinary tract infections
Source: J Clin Lab Anal. 2022 Mar 29;36(5):e24399. doi: 10.1002/jcla.24399 (PMC9102756; doi:10.1002/jcla.24399)
Supplement: Supplementary file 1 — Table S1 [file JCLA-36-e24399-s001.docx]

| Additional file 1 Results of the in vitro susceptibility testing of M. morganii isolates against various antibiotics (S = susceptible, R = resistant，I= intermediate) | | | | | | | | | | | |
| --- | --- | --- | --- | --- | --- | --- | --- | --- | --- | --- | --- |
|  | Case 1 | Case 2 | Case 3 | Case 4 | Case 5 | Case 6 | Case 7 | Case 8 | Case 9 | Case 10 | Case 11 |
| Colony count (CFU/ml） | ≥10^5 | 2× 10^4 | 1.5 × 10^4 | ≥ 10^5 | ≥ 10^5 | 2 × 10^4 | 7 × 10^4 | ≥ 10^5 | ≥ 10^5 | ≥ 10^5 | ≥ 10^5 |
| Amikacin | S | S | R | S | S | S | S | S | S | S | S |
| Ampicillin/sulbactam | S | I | R | R | R | I | R | R | I | R | R |
| Aztreonam | S | S | S | S | S | S | S | S | S | S | S |
| Ertapenem | S | S | S | S | S | S | S | S | S | S | S |
| Trimethoprim/sulfamethoxazole | S | S | R | S | S | S | S | R | R | R | S |
| Ciprofloxacin | S | S | R | S | S | S | S | R | S | R | S |
| Fosfomycin | / | / | / | / | I | / | / | I | R | R | / |
| Chloramphenicol | / | / | / | / | S | / | / | R | R | I | / |
| Meropenem | S | S | S | S | S | S | S | S | S | S | S |
| Minocyline | / | / | R | / | S | / | / | / | S | / | / |
| Moxifloxacin | S | S | R | S | S | S | S | I | S | R | S |
| Nalidixic Acid | S | S | R | S | S | S | S | R | S | R | S |
| Norfloxacin | S | S | R | S | S | S | S | I | S | S | S |
| Piperacillin | S | S | R | S | S | S | S | S | S | I | S |
| Piperacillin/Tazobactam | S | S | S | S | S | S | S | S | S | S | S |
| Gentamicin | S | S | R | S | S | S | S | R | S | R | S |
| Ticarcillin | / | S | R | S | S | S | S | I | S | S | S |
| Ticarcillin/Clavulanic acid | / | / | / | S | S | / | S | S | S | S | S |
| Cefepime | S | S | S | S | S | S | S | S | S | S | S |
| Cefuroxime Axetil | R | R | R | R | R | R | R | R | R | R | R |
| Cefoperazone/Sulbactam | / | / | S | S | S | / | / | S | S | S | / |
| Ceftriaxone | S | S | R | S | S | S | S | S | S | S | S |
| Cefalotin | R | R | R | R | R | R | R | R | R | R | R |
| Cefotaxime | S | S | R | S | S | S | S | S | S | S | S |
| Ceftazidime | S | S | S | S | S | S | S | S | S | S | S |
| Cefotetan | / | S | S | S | S | S | S | S | S | S | S |
| Cefazolin | R | / | R | / | R | / | / | / | / | / | / |
| Ceftizoxime | S | S | I | S | R | S | I | I | S | I | I |
| Tobramycin | S | S | R | S | S | S | S | R | S | S | S |
| Imipenem | S | / | R | R | R | R | S | / | I | S | S |
| Levofloxacin | S | S | R | S | S | S | S | R | S | I | S |
| Amoxicillin /Clavulanate Potassium | R | / | / | / | / | / | / | / | / | / | / |
| Ampicillin | R | / | / | / | / | / | / | / | / | / | / |
| Cefuroxime | R | / | / | / | / | / | / | / | / | / | / |
| Nitrofurantoin | R | / | / | / | / | / | / | / | / | / | / |
| Tetracycline | R | / | / | / | / | / | / | / | / | / | / |
| Tigecycline | R | / | / | / | / | / | / | / | / | / | / |
